# Validation of Droplet Digital Polymerase Chain Reaction for *Salmonella* spp. Quantification

**DOI:** 10.3389/fmicb.2020.01512

**Published:** 2020-07-07

**Authors:** Carolina Villamil, Martha Nancy Calderon, Maria Mercedes Arias, John Emerson Leguizamon

**Affiliations:** ^1^Departamento de Química, Universidad Nacional de Colombia, Bogota, Colombia; ^2^Grupo de Metrología en Bioanálisis, Instituto Nacional de Metrología, Bogota, Colombia

**Keywords:** droplet digital PCR, *Salmonella*, validation, food safety, bioanalysis

## Abstract

Salmonellosis is a foodborne disease caused by *Salmonella* spp. Although cell culture is the gold standard for its identification, validated molecular methods are becoming an alternative, because of their rapidity, selectivity, and specificity. A simplex and duplex droplet digital polymerase chain reaction (ddPCR)-based method for the identification and quantification of *Salmonella* using *ttr*, *invA, hilA, spaQ*, and *siiA* gene sequences was validated. The method has high specificity, working interval between 8 and 8,000 cp/μL in ddPCR reaction, a limit of detection of 0.5 copies/μL, and precision ranging between 5 and 10% measured as a repeatability standard deviation. The relative standard measurement uncertainty was between 2 and 12%. This tool will improve food safety in national consumption products and will increase the competitiveness in agricultural product trade.

## Introduction

*Salmonella* spp. is one of the most important pathogens and the leading cause of foodborne diseases (World Health Organization, 2015). Hence, the development and validation of biometric tools, such as measurement methods and reference materials, are important for making decisions related to the national and global trade of food products (European Comission, 2007), as well as for public health surveillance, among others.

With recent advances in molecular techniques, several rapid methodologies for the detection and quantification of pathogens based on specific genes and proteins have been developed. Besides their rapidity, these methods provide lower limits of detection and better specificity (Law et al., 2014). In particular, the detection of *Salmonella* spp. using polymerase chain reaction (PCR) is based on the presence of single genes in a large number of different serotypes and unique copies within the genome. Among the most commonly used ones are *(i) invA*, which encodes for invA, a protein required for invasion of the bacterium to epithelial cells (Galan et al., 1992), *(ii) hilA* (hyperinvasive locus), which encodes for hilA, a transcriptional regulator of the OmpR/ToxR family that activates the expression of genes of the type III secretion system, required for bacterial invasion (Boddicker et al., 2003), *(iii)* the *ttr* locus, which is required for respiration with tetrathionate and survival of the bacteria after infection (Law et al., 2014), *(iv) spaQ*, a part of the inv/spa complex, which is required for the entry of the bacteria into non-phagocytic host cells (Kurowski et al., 2002), and *(v) siiA*, which produces a regulatory protein encoded by SP14, important for adhesion to epithelial cells during *Salmonella* invasion (Zhao et al., 2017).

Digital PCR is considered a potential primary method of measurement as it does not require standards of the same quantity to yield a measurement result (Gutierrez-Aguirre et al., 2015). It is based on microfluidics technology, which allows the generation of multiple reaction partitions (1,000–10 million, depending on the platform) that work as individual reactions. Based on the positive or negative fraction and following the Poisson distribution, it is possible to determine the absolute concentration of the target of interest in terms of the number of copies per microliter (cp/μL) in the dPCR reaction (Huggett et al., 2013; Morisset et al., 2013; Magnusson and Örnemark, 2014). Besides, owing to the dilution and partitioning of the sample, the technique is less sensitive to inhibitors, which results in better precision and less uncertainty contribution (Somanath and Kerry, 2016). In this study, an in-house validation has been described for droplet digital PCR (ddPCR) for quantitating genomic DNA from *Salmonella* spp. using five different targets, run in two duplex and one simplex form. The performance characteristics evaluated were specificity, working interval, precision, limit of detection (LOD), limit of quantification (LOQ), and measurement uncertainty, contributing to the strengthening of monitoring process of *Salmonella* in agricultural products in Colombia.

## Materials and Methods

### Bacterial Strains and Growth Conditions

Sixteen serotypes of *Salmonella enterica* subsp. *enterica* and other reference strains phylogenetically close to *Salmonella* spp., such as *Escherichia coli, Klebsiella* spp., and *Shigella* spp. (Table 1) were cultured in solid tryptone soya agar (TSA) or XLT4 (xylose, lysine, and tergitol 4) and liquid selective medium Rappaport Vassiliadis (RV). All strains were grown at 37°C for 12–18 h in liquid medium RV before DNA extraction.

**TABLE 1:** Enterobacteria strains used in this study.

Strain description	Source
*Salmonella enteritidis*	ATCC^®^ 13076
*Salmonella typhimurium*	Clinical isolate
*Salmonella typhi*	Clinical isolate
*Salmonella derby*	Clinical isolate
*Salmonella dublin*	Clinical isolate
*Salmonella give*	Clinical isolate
*Salmonella saintpaul*	Clinical isolate
*Salmonella hadar*	Clinical isolate
*Salmonella infantis*	Clinical isolate
*Salmonella anatum*	Clinical isolate
*Salmonella panama*	Clinical isolate
*Salmonella typhimurium var5-*	Clinical isolate
*Salmonella javiana*	Clinical isolate
*Salmonella braenderup*	Clinical isolate
*Salmonella muenchen*	Clinical isolate
*Salmonella paratyphi A*	Clinical isolate
*Bacillus cereus*	ATCC^®^ 10876
*Enterococcus faecalis*	ATCC^®^ 14506
*Escherichia coli O104:H4*	ATCC^®^ BAA-2326
*Escherichia coli O145:NM*	ATCC^®^ CDC99–3311
*Escherichia coli O157:H7*	ATCC^®^ 35150
*Proteus mirabilis*	ATCC^®^ 12453
*Proteus vulgaris*	ATCC^®^ 33420
*Shigella boydii*	ATCC^®^ 9207
*Shigella sonnei*	ATCC^®^ 9290
*Staphylococcus aureus*	ATCC^®^ 25923
*Staphylococcus aureus*	ATCC^®^ 6538
*Vibrio parahaemolyticus*	ATCC^®^ 17802

*ATCC, Reference strain from the American Type Culture Collection.*

### DNA Extraction

A protocol equally efficient for both Gram-positive and Gram-negative bacteria was used with some modifications (Atashpaz et al., 2010). Briefly, 3 mL cell culture in the exponential phase was centrifuged at 5,000 rpm for 5 min at 4°C. Then, 800 μL lysis buffer [CTAB 2% (w/v), Tris−HCl 100 mM, NaCl 1.4 M, EDTA 20 mM, and LiCl 0.2% (w/v), pH 8.0] was added to the bacterial pellet. The samples were incubated at 65°C for 30 min for Gram-negative bacteria and 2 h for Gram-positive bacteria. Then, these were centrifuged at 10,000 rpm for 5 min at 4°C. The supernatant was transferred to a new tube where 1 volume of chloroform-isoamyl alcohol (24:1) was added to precipitate the proteins. The samples were gently mixed and centrifuged at 12,000 rpm for 8 min at 4°C. Two further extractions with chloroform-isoamyl alcohol (24:1) were performed on the aqueous phases for each sample. From the pooled supernatants, the DNA was precipitated by the addition of 1 volume of cold isopropanol and 100 μL of 5 M sodium acetate after overnight incubation at −20°C. Subsequently, the samples were centrifuged at 12,000 rpm for 15 min at 4°C and washed with 1 mL of 70% ethanol by inverting the tubes. Finally, once the ethanol had evaporated, the DNA was resuspended in 100 μL 1X TE buffer (Tris–HCl 10 mM, EDTA 1 mM pH 8.0) and stored at −20°C. The quality of the extracted genomic DNA was evaluated using UV spectrophotometry by measuring the absorbance ratios at 260 nm/280 nm and 260 nm/230 nm; DNA integrity was assessed using electrophoresis on 1% agarose gel.

### Primers and Probes

The primers and probes (Biosearch Technologies, Petaluma, CA, United States, purified by HPLC) for each target are shown in Table 2.

**TABLE 2:** Primers and probes selected for the analysis of *Salmonella spp.*

Gene	Name	Sequence 5′-3′	Amplicon sequence	Amplicon size (pb)	References
*invA*	invA_176F	CAACGTTTCCTGCGGTACTGT	CAACGTTTCCTGCGGTACTGTTAATTAC	119	González-Escalona et al., 2012
	invA_291R	CCCGAACGTGGCGATAATT	CACGCTCTTTCGTCTGGCATTATCG		
	invA_Tx_208 TXa-	FAM-CTCTTTCGTCTGGCATTATC GATCAGTACCA-BHQ1	ATCAGTACCAGTCGTCTTATCTT GATTGAAGCCGATGCCGGTGAAAT TATCGCCACGTTCGGGCAA		

*ttr*	ttr6F4287 – Directo	CTCACCAGGAGATTACAACATGG	CTCACCAGGAGATTACAACATGG	95	Rothrock et al., 2013;
	ttr4R4381 – Reverso	AGCTCAGACCAAAAGTGACCATC	CTAATTTAACCCGTCGTCAGTGGCTA		Ssemanda et al., 2018;
	ttr5p4336 – Sonda	CFO-CACCGACGGCGAGACCG ACTTT-BHQ1	AAAGTCGGTCTCGCCGTCGGTG GGATGGTCACTTTTGGTCTGAGCT		Boer et al., 2019

*siiA*	siiA Fw	ACGACTGGGATATGAACGGGGAA	ACGACTGGGATATGAACGGGGAATT	107	Ben Hassena et al., 2015
	siiA Rv	TCGTTGTACTTGATGCTGCGGAG	ATTTTAATGAAAGAGATTAAG		
	siiaA Tr	FAM-ATCCTGATGTAGTTATTGAC ATGAG-BHQ1*	AAGATATATCCTGATGTA GTTATTGACATGAGTGTTAACT CCGCAGCATCAAGTACAACGA		

*hilA*	hilA F	ACTGTACGGACAGGGCTAT*	ACTGTACGGACAGGGCTATCGGTT	129	McCabe et al., 2011;
	hilA R	AGA CTC TCG GAT TGA ACC TGA-3′	TAATCGTCCGGTCGTAGTGGTGT		Wang et al., 2016
	hilA Lc640	HEX-TCGTCCGGTCGTAGTGGTG TCTCC-BHQ1	CTCCGCCAGCGCCGCAACCTACGACT CATACATTGGCGATACTTCCTTTTCAGAT GCAGGATCAGGTTCAATCCGAGAGTCT		

*spaQ*	spaQ Fw	CCTGACGCCCGTAAGAGA	CCTGACGCCCGTAAGAGAGTAAAACTTA	113	Kurowski et al., 2002;
	spaQ Rv	GCAATTACAGGAACAGACGCT	CGCCATACCAGCCAGACAGTAAA		Ekiri et al., 2016
	spaQ P	FAM-TAAAACTTCGCCATACCAGC CAGACA-BHQ1	ACAAGCATAAACACACGCCA AGTAATTTAATGCCAAAAGG CAGCGTCTGTTCCTGTAATTGC		

FAM: 6-Carboxyfluorescein, HEX: Hexachlorofluorescein, CFO: Cal Fluor Orange 560, equivalent to HEX dye. BHQ1: Black Hole Quencher 1. *Modified in this study from references.

### Quantitative PCR (qPCR)

Quantitative PCR was used as a preliminary test for evaluating the reaction efficiency of primers and probes for each target in simplex mode (CFX 96 Deep well- BioRad, cat. 1855196). Six dilutions of *S. enteritidis* reference strain ATCC 13076 DNA in 1X TE buffer were prepared (100–0.01 ng/μL). The 20 μL reaction contained 1X iTaq^TM^ Universal Probes Supermix (BioRad cat. 1725131), 400 nM primers, 300 nM probes, and nuclease-free water. 1X TE was used as a non-template control (NTC). All samples were run in triplicate. The amplification cycle consisted of initial denaturation at 95°C for 10 min, followed by 40 cycles of denaturation at 95°C for 15 s and annealing-elongation at 60°C for 30 s, with a heating ramp of 2°C/s. Efficiency was evaluated using Eq. 1,

(1) $$E=10^{\frac{-1}{m}}-1$$

where *E* is the efficiency and *m* the slope of a linear regression between the amplification cycle and the log concentration of each sample. The acceptability criterion for PCR efficiency was 90–100%.

### ddPCR

In order to establish the best conditions to perform the validation assay for every target sequence, the annealing temperature through a gradient from 55–63°C was evaluated. In addition, the primer concentration was also evaluated from 300 to 900 nM, and the ramp rate between 1 and 2°C/s. The aim was to establish general conditions to amplify the target sequences under the same experimental conditions. After optimization, the best amplification temperature was 60°C with a ramp rate of 2°C/s and 600 nM concentration of primers.

After optimization, according to MIQE guidelines (Huggett et al., 2013; Supplementary Table S8), ddPCR assay was performed in simplex (*spaQ*), and duplex (*hilA-siiA* and *invA*-ttr) form: *(i)* according to the number of total reactions, a stock master mix with 1X ddPCR^TM^ Supermix for Probes (BioRad, CA, United States cat. 1863024), 600 nM primers, 300 nM probes, and nuclease-free water was prepared; *(ii)* for every triplicate 60 μL of the stock master mix was weighed, *(iii)* and finally 6 μL of DNA template (genomic DNA of *S. enteritidis*, measured gravimetrically) were added to complete 66 μL. Then to avoid pipetting bias, 21 μL PCR reaction mixture and 70 μL generator oil (BioRad cat. 183005) were loaded into an 8-well DG8 cartridge (BioRad cat. 1864008), for every replicate, in order to have the same dilution factor. Droplets were generated in a QX200 droplet generator, transferred to a 96-well plate (BioRad cat. 12001925), and sealed with a PX1 PCR plate sealer (BioRad cat. 1814000). Reactions were amplified in a CFX96 deep-well thermocycler (BioRad cat. 185–5196), and the cycling conditions were: 95°C for 10 min, 40 cycles at 95°C for 30 s, and 60°C for 1 min, with an overall ramp rate of 2°C/s. All the reactions were read in the QX200 droplet reader system (two detection channels FAM/EvaGreen and VIC/HEX) using the Quantasoft^TM^ software V1.7 from BioRad.

### Data Acquisition and Analysis

Droplets were classified as PCR-positive or PCR-negative according to a threshold fluorescence value set manually using the Quantasoft^TM^ software V1.7. Technical reasons for excluding data from the analysis were: *(i)* the total number of droplets <12,000 (BioRad, 2018) and *(ii)* wells with amplitude different from those of other wells with the same target. This variance indicated poor droplet generation, possibly due to poor handling or mixing of samples, and is likely to generate erroneous concentration values. Reference material ERM-AD623 (White et al., 2015) was used as the amplification control for assessing the performance of the thermocycling parameters.

Data were exported to a spreadsheet to calculate the concentration of the sample in cp/μL in the ddPCR reaction, with a droplet volume of 0.819 ± 0.017 nL (*k* = 2; Eqs 2, 3), as long as the confidence limits were associated with Poisson distribution.

(2) $$C_{\text{sample}}=\frac{\lambda }{v\times D}$$

where:

(3) $$\lambda =-In\left(\frac{N}{P}\right)$$

*C*_*sample*_: Sample concentration $\left(\frac{cp}{uL}\right)$

_λ_ : Copies/partition

*N*: Number of negative partitions

*P*: Number of total partitions

*v*: Droplet volume

*D*: Total dilution of the sample from the master mix.

The droplet volume was calculated as the mean of values reported previously (Corbisier et al., 2015; Dagata et al., 2016) and the uncertainty was estimated based on a rectangular distribution, to encompass any influence of droplet volume variability and to avoid underestimation of measurement uncertainty (Košir et al., 2017; Emslie et al., 2019).

### Method Performance Characteristics

The performance characteristics evaluated were specificity, working interval, equivalence between simplex and duplex assay, precision as repeatability and intermediate precision, the LOD, the LOQ, and measurement uncertainty.

## Results

### qPCR Amplification Efficiency of DNA Targets

From the optimized extraction process, 1 mL of 4430 ng/μL of DNA was obtained, 1:4 dilution was prepared as a working solution (WS; see Supplementary Figure S1). Preliminary tests using qPCR were performed to determine whether all gene sequences amplified correctly and whether PCR inhibitors were present. The qPCR amplification efficiencies calculated for the five targets were between 90 and 99% (Table 3), indicating that inhibitors were not present in the starting genomic DNA. The dilutions and log concentration showed a good correlation, indicating the suitability of qPCR for the studied targets.

**TABLE 3:** Amplification efficiency for target sequences under study.

Gene	Slope	Efficiency (%)	*R*^2^
*invA*	–3.346	99.0	1.000
*ttr*	–3.358	98.5	1.000
*siiA*	–3.383	97.5	1.000
*hilA*	–3.587	90.0	0.980
*spaQ*	–3.361	98.4	1.000

### Specificity

The DNA of sixteen serotypes of *Salmonella enterica* subsp. *enterica* (Table 1) was amplified using the optimized ddPCR method. On the other hand, the DNA of four enterobacteria groups closely related and not related to the species under study were also evaluated. Each group contained 1 ng/μL DNA of each species, and they were classified as *(i)* Gram-positive bacteria group: *Staphylococcus aureus* subsp. *aureus, Bacillus cereus, and Enterococcus faecalis; (ii) Shigella* group*: Shigella sonnei, Shigella boydii*, and *(iii)* SHEC *Escherichia* Group: *E. coli* O104: H4, *E. coli* O145: NM, *E. coli* O157: H7. As a positive control for this assay, a DNA mixture of 1 ng/μL of four serotypes of *Salmonella* spp*., (S. typhimurium, S. typhi, S. derby*, and *S. paratyphi* A.) was used.

The specificity of the selected sequences was evaluated initially using an *in silico* analysis. Then, sixteen serotypes of *Salmonella* spp. (Table 4) were evaluated using a ddPCR assay. The results indicated that a positive amplification response was generated for all *Salmonella* serotypes and that negative amplification results were generated for all Enterobacteria strains evaluated.

**TABLE 4:** Specificity analysis.

Serovar	Results for all targets
*Bacillus cereus*	−
*Enterococcus faecalis*	−
*Escherichia coli O104:H4*	−
*Escherichia coli O145:NM*	−
*Escherichia. coli O157:H7*	−
*Proteus mirabilis*	−
*Proteus vulgaris*	−
*Shigella boydii*	−
*Shigella sonnei*	−
*Staphylococcus aureus subsp. aureus*	−
*Staphylococcus aureus subsp. aureus*	−
*Vibrio parahaemolyticus*	−
*Salmonella typhimurium*	*+*
*Salmonella typhi*	*+*
*Salmonella derby*	*+*
*Salmonella enteritidis*	*+*
*Salmonella dublin*	*+*
*Salmonella give*	*+*
*Salmonella saintpaul*	*+*
*Salmonella hadar*	*+*
*Salmonella infantis*	*+*
*Salmonella anatum*	*+*
*Salmonella panama*	*+*
*Salmonella typhimurium var5-*	*+*
*Salmonella javiana*	*+*
*Salmonella braenderup*	*+*
*Salmonella muenchen*	*+*
*Salmonella paratyphi A*	*+*

### Working Interval

**FIGURE 1:**
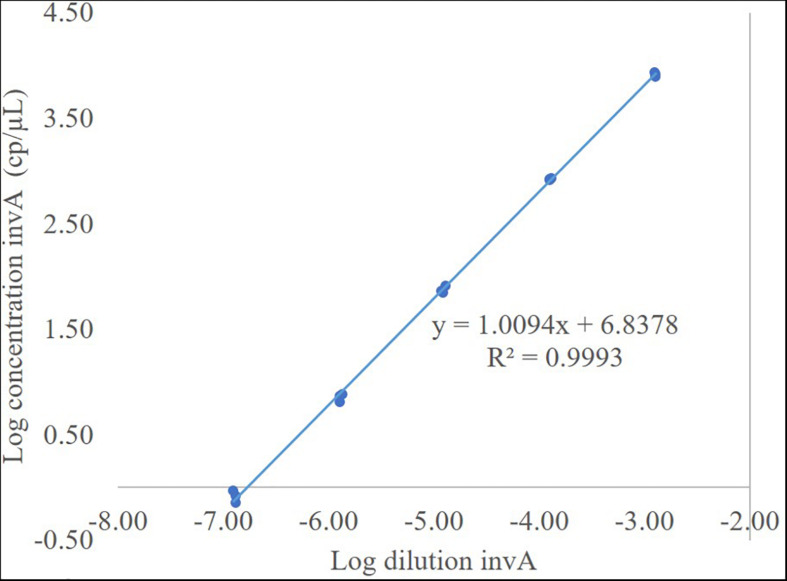
The working interval for *invA*. Total DNA gravimetric dilution in Log scale vs. DNA concentration (cp/μL) in Log scale.

Using *S. enteritidis* ATCC 13076 DNA, five WS from 10 to 0.001 ng/μL (as nominal concentration), were prepared. Serial gravimetric dilution from WS to ddPCR master mix reaction were measured in triplicate on three different days (see Supplementary Tables S1–S5). The working interval was determined using least squares regression analysis of the linear relationship between each dilution and their corresponding concentration (cp/μL) using the ddPCR method (Table 5). Dilution value is used to analyze the working interval as the nominal concentration is just an estimate of the real concentration. An example is presented in Figure 1. Grubbs’ test was performed to determine outliers on one *hilA* level replicate. The results for the NTC were negative.

**TABLE 5:** Regression analysis for each target sequence evaluated at 95% confidence.

Gene	Slope	Intercept	*R*^2^
*invA*	1.0094	6.8378	0.9996
*ttr*	1.0200	6.8474	0.9990
*spaQ*	0.9749	6.2832	0.9985
*hilA*	0.9707	6.2528	0.9985
siiA*	1.0063	6.3995	0.9996

*siiA shows good linearity up to fourth Log dilution.

The acceptance criteria for linearity were as follows: a slope statistically different from zero and correlation coefficient >0.99 (Deprez et al., 2012). The method showed excellent linearity between 1 and 8,000 cp/μL in reaction (except for *siiA*, the interval of which was from 8 to 8,000 cp/μL). Therefore, the working interval for the whole method was established as 8–8,000 cp/μL.

**FIGURE 2:**
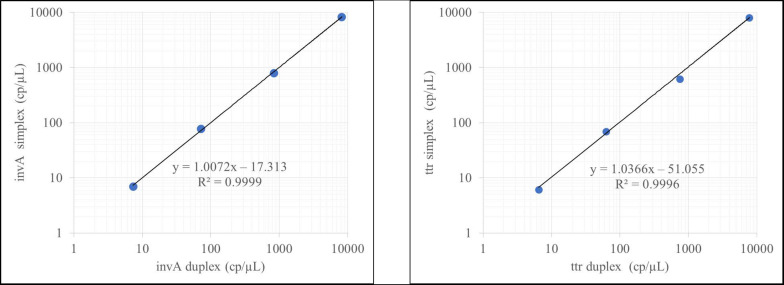
Duplex evaluation for the *invA-ttr* assay.

As part of the validation process, the ability of the method to run in a duplex form (*invA-ttr* and *hilA- siiA*) was also evaluated through regression analysis. Both methods were compared over the working interval, using data of four concentration levels (8–8,000 cp/μL) in triplicate and on three different days (Figure 2; see Supplementary Table S6). A slope statistically equal to 1 and correlation coefficient >0.99 were set as acceptance criteria.

According to Figure 2, the results obtained did not differ when *invA* and *ttr* were measured independently or in combination. Identical results were obtained for *hilA* and *siiA* target sequences (see Supplementary Figure S2). This allowed simultaneous evaluation of four different targets in two duplex reactions with the same confidence.

**FIGURE 3:**
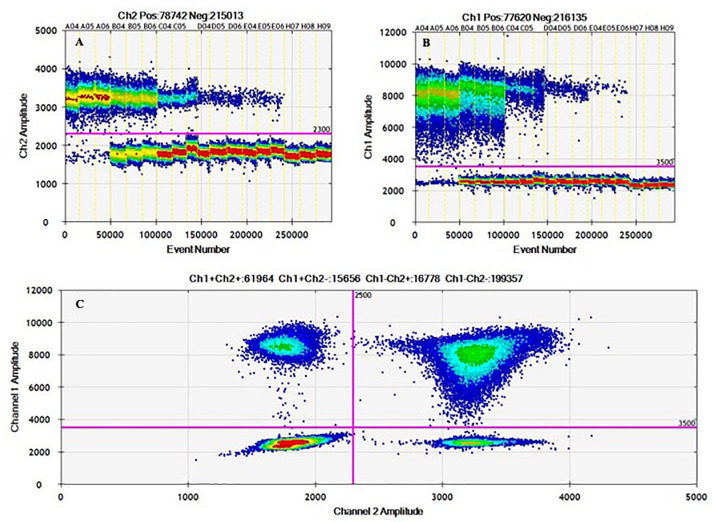
1D and 2D ddPCR heat maps from working interval evaluation for duplex mode using hila – siiA targets. Evaluation of the working interval for hilA **(A)** and siiA **(B)** in the 1D plot (droplets vs. fluorescence amplitude), and 2D plot (channel one fluorescence (FAM) vs. channel two fluorescence (HEX) for each droplet; **(C)**.

In addition, the amplitude heat plots of 1D and 2D duplex reactions showed clear populations of droplet distribution in positive and negative partitions, minimizing misclassification of droplets (rain) and absence of cross-reaction (Figure 3). As DNA distribution follows a random pattern, in the 2D plot, droplets are cluster in four groups: *(i)* double negatives, *(ii)* FAM positives and HEX negatives, *(iii)* FAM negatives and HEX positives, or *(iv)* double positives; not all positive partitions are double-positive in the duplex assay (Figure 3C) due to some possible fragmentation process that affect DNA integrity (Furuta-Hanawa et al., 2019); Table 6 shows the variation of clusters fraction with DNA concentration.

**TABLE 6:** Relative fraction in respect to total partitions for every cluster in the duplex amplification for *hilA-siiA* targets.

**Concentration level**	***hilA*+ *siiA*+** **cluster**	**Relative fraction**	***hilA*+ *siiA*- cluster**	Relative fraction	*hilA*- *siiA*+ cluster	Relative fraction	*hilA*- *siiA*- cluster	Relative fraction	Total partitions	Ratio *hilA*/*siiA*
1	50,162	99,6%	78	0,2%	125	0,2%	0	0,0%	50,365	0.93
2	11,582	22,5%	12,485	24,3%	13,290	25,8%	14,072	27,4%	51,429	0.96
3	215	0,5%	2,680	6,0%	2,878	6,4%	38,851	87,1%	44,624	0.93
4	5	0,0%	357	0,7%	340	0,7%	49,054	98,6%	49,756	1.05

### Precision

Five concentration levels covering 1–8,000 cp/μL in ddPCR reaction, in triplicate reactions on three different days (see Supplementary Tables S1–S5), relative repeatability standard deviation (Eq. 4), and relative intermediate standard deviation (Eq. 5), were calculated using one factor ANOVA were calculated using one-factor ANOVA (Deprez et al., 2016).

(4) $$S_{repeat,rel}=\frac{\sqrt{MS_{witinrun}}}{C_{sample,mean}}$$

(5) $$S_{interm,rel}=\frac{\frac{\sqrt{MS_{betweenrun}-MS_{withinrun}}}{n_{replicates}}}{C_{sample,mean}}$$

where

*MS_witin run_* : Within run mean squares calculated using one-way ANOVA

*MS_between run_* : Between run mean squares calculated using one-way ANOVA

*n*_*replicates*_: Number of replicates per run

*C*_*sample, mean*_: Averaged copy number concentration calculated over all runs.

**FIGURE 4:**
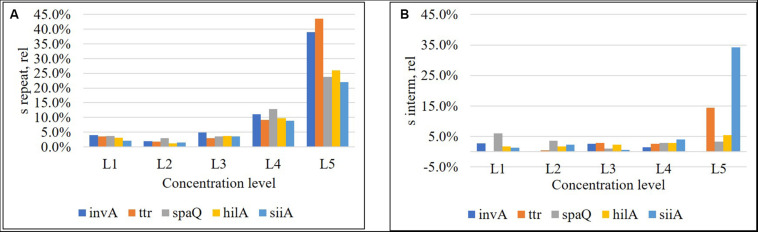
Relative repeatability standard deviation **(A)** and relative intermediate standard deviation **(B)** of the method for each target sequence. Concentration levels were, L1: 8,000 cp/μL, L2: 800 cp/μL, L3: 80 cp/μL, L4: 8 cp/μL, and L5: 1 cp/μL.

Repeatability was below 5% for the three highest concentration levels and increased to 10% for the fourth one. However, for the lowest concentration (approximately 1 cp/μL in reaction), repeatability, was beyond 20% (Figure 4A), while intermediate precision was below 5% in almost the entire interval, except for *ttr*, and *siiA* (Figure 4B). When MS between <MS within, the *S*_*interm, rel*_ was considered negligible with respect to *S*_*repeat*,*rel*_.

### LOQ

Limit of quantification represents the lowest copy number of the analyte that can be determined with acceptable performance. We have defined the performance criteria as relative repeatability standard deviation <20%, according to the precision study. The higher values represent a significant increase in the measurement uncertainty. Thus, the LOQ based on the response of all the genes in this study was established as 8 cp/μL (Figure 4A).

### LOD

**FIGURE 5:**
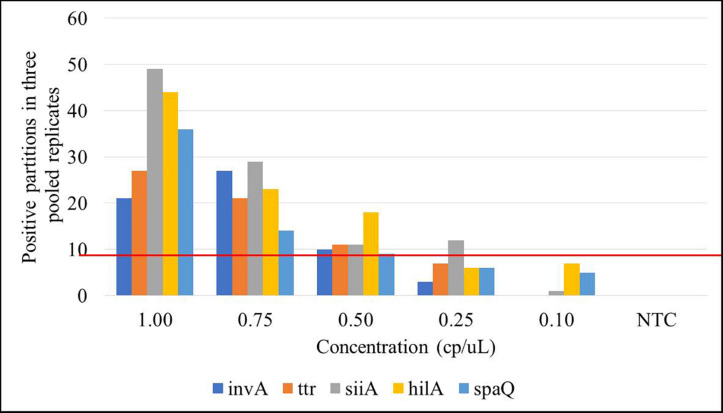
Limit of detection (LOD) for the targets under study. Each bar represents the positive partitions over the three pooled replicates within the 5 concentration levels (L1: 8,000 cp/μL, L2: 800 cp/μL, L3: 80 cp/μL, L4: 8 cp/μL, and L5: 1 cp/μL) compared to the no template control (NTC). Red line represents the selected threshold: nine positive partitions.

Limit of detection is defined as the lowest analyte concentration that can be distinguished from zero with a specified level of confidence. LOD was determined as the lowest concentration level, where at least three positive droplets were present in all three replicates (Morisset et al., 2013), or equivalent in at least nine positive partitions in the pooled replicates. Amplifications were performed in a simplex mode for *spaQ* and duplex mode for *invA-ttr* and *hilA-siiA*. Five gravimetric serial dilutions (1–0.1 cp/μL in the ddPCR reaction) were prepared and run in triplicate. Figure 5 presents the results in which each bar represents the total positive partitions obtained for three replicates compared to a total negative NTC. The estimated LOD was 0.5 cp/μL in the ddPCR reaction for three positive droplets (on average) in at least 12,000 total partitions.

### Measurement Uncertainty

**FIGURE 6:**
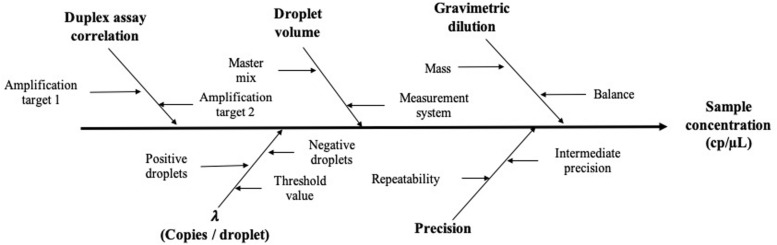
Schematic representation of the main factors affecting the measurement value and its uncertainty.

Based on precision data and considering the mathematical model, the measurement uncertainty estimation was established: first, all possible sources of uncertainty were considered (Figure 6), and they were grouped according to their relation and quantified. Then, all contributions were combined to have standard uncertainty (Eq. 6; Joint Committee for Guides in Metrology [JCGM], 2008).

(6) $$u_{sample}=\sqrt{u_{Model}^{2}+u_{Precision}^{2}}$$

where

$u_{Model}^{2}$ corresponds to the uncertainty contribution provided by the mathematical model (Eq. 2) of the measuring method and includes the uncertainty provided by the partitioning of the sample (λ factor), gravimetric dilution, and droplet volume (Eq. 7),

(7) $$u_{Model}=C_{sample}\times \sqrt{{\left(\frac{u_{\lambda }}{\lambda }\right)}^{2}+{\left(\frac{u_{D}}{D}\right)}^{2}+{\left(\frac{u_{v}}{v}\right)}^{2}}$$

and $u_{Precision}^{2}$, which corresponds to the precision uncertainty provided by the total variation of each concentration level, was defined as the highest degree of dispersion obtained between replicates $s_{repeat}^{2}$ and between different days $s_{interm}^{2}$ (Eq. 8),

(8) $$u_{Precision}=\sqrt{\frac{s_{repeat}^{2}+s_{interm}^{2}}{n}}$$

*n*: Days of measurement.

**FIGURE 7:**
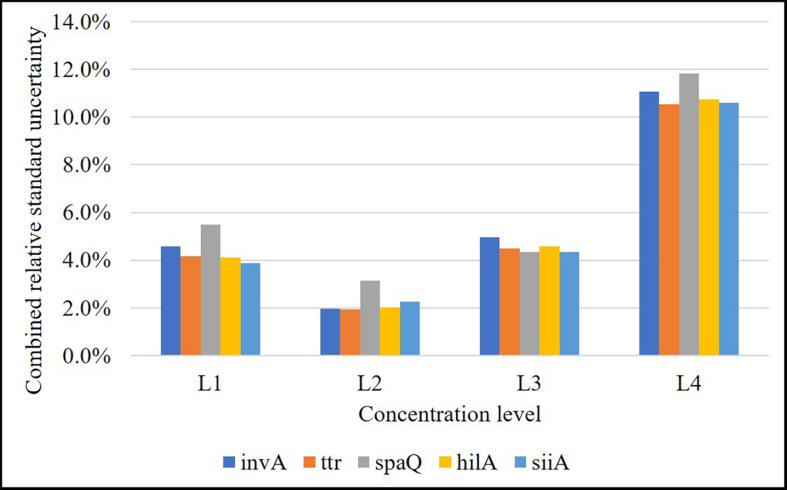
Relative combined uncertainty for each target in each concentration level over the working range (L1: 8,000 cp/μL, L2: 800 cp/μL, L3: 80 cp/μL, and L4: 8 cp/μL in reaction). Level 2 is the one with the lowest uncertainty (approximately 2%).

According to the validation data for all target sequences, the relative measurement uncertainty associated with the mathematical model varied between 1,6 and 9% (Table 7). The maximum contribution corresponded to λ factor (50–99%) at all levels (see Supplementary Figure S3). After combining the mathematical model and measurement uncertainty contributions (Eq. 6), the combined uncertainty was calculated for every target in every concentration level (Figure 7). The relative standard uncertainty value ranged between 2 and 12%, while its lower value was obtained at the concentration of 800 cp/μL in the ddPCR reaction.

**TABLE 7:** Relative measurement uncertainty using the mathematical model for the five targets.

Level	Concentration (cp/uL)	Relative uncertainty (%)
1	8,000	3.6
2	800	1.6
3	80	3.8
4	8	9.0

*Data correspond to an average value.*

### Multiplex Correlation Analysis

The correlation between the targets *invA-ttr* and *hilA-siiA* was evaluated to determine the contribution source to the uncertainty associated with covariance between target sequences in duplex assays. Considering that all target sequences are single copy, the correlation was evaluated, keeping the primers concentration constant for one target sequence (600 nM), while this was varied for the other one as 0, 150, 300, 450, 600, 750, and 900 nM. In all cases, the probe concentration was 300 nM. The slope and correlation coefficients were calculated and their significance analyzed using the student’s *t*-test with a 95% confidence.

**FIGURE 8:**
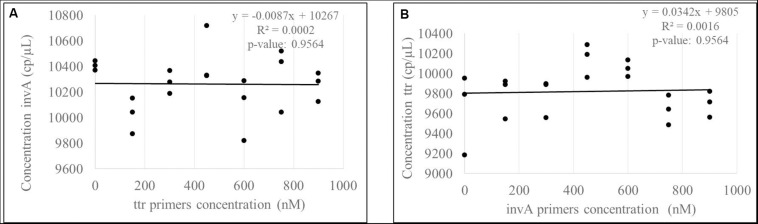
Evaluation of correlation between *invA* **(A)** *-ttr* **(B)** multiplex amplification. According to *p*-values, there are not a significant correlation between targets.

As the slopes were statistically equal to zero and the correlation were not significant (Figure 8 and Supplementary Figure S4) for any of the target sequences, then, there is not a covariance contribution to the combined uncertainty (Joint Committee for Guides in Metrology [JCGM], 2008).

## Discussion

Digital PCR is a relatively new and promising technology, which is currently being used for detecting pathogens in food (Quan et al., 2018). The molecular quantification of specific genes in food samples is a useful tool for evaluating the quality and safety of food products in time-, effort-, and cost-effective manner to generate reliable results (Huber, 2007; Morisset et al., 2013). At the same time, digital PCR is considered a potential primary method of measurement in chemical metrology, allowing among its multiple applications, developing reference materials in bioanalysis especially at the clinical and industrial level (Whale et al., 2018).

The method reported here uses five targets of common use for the identification of *Salmonella* spp. using ddPCR. Two assays in duplex mode (*invA-ttr* and *hilA-siiA*) and one in simplex mode (*spaQ*) were used. The qPCR dilution curves showed that the DNA extraction and purification processes were suitable for obtaining DNA of good quality, which is necessary for validation. The amplification efficiency confirmed the absence of inhibitors that can affect the digital PCR validation process.

2D plots for duplex amplification (Figure 3), shows there is an apparent DNA integrity change, and some variation with DNA concentration. From Table 6, most positive droplets are double-positive for both target sequences (99.6%) at 8,000 cp/μL, but this proportion decrease with concentration level, passing to 22.5 and 0.5% in 2 log DNA concentrations (Table 6 and Supplementary Table S7), indicating a possible fragmentation process in the DNA. The observed clusters relative fraction depends on the fraction of positive droplets. For a fraction higher than 90%, according to Eq. 3, there will be 2 or more copies per partition while for lower ratios, there will be lower occupancy degree of DNA molecules. Droplets with less amplitude for the double positive cluster could indicate an imbalanced amplification process between hilA and siiA DNA target sequences for a non-competing duplex reaction (Whale et al., 2016). However, the ration between hilA/siiA and invA/ttr target sequences keeps around 1, indicating that neither this fragmentation process, nor the imbalance amplification process are enough to affect the quantification in duplex form by digital PCR.

Although four targets (*invA, ttr, hilA*, and *spaQ*) showed good linearity over 5 log concentrations (covering 1 cp/μL, according to linear regression analysis), the working interval goes from 8,000 to 8 cp/μL in ddPCR reaction, where the lowest concentration corresponded to the LOQ, which was defined based on precision criteria of maximum 20% as a relative repeatability standard deviation. Precision values higher than 20% (as they were obtained for the lowest levels), contributed more to combined measurement uncertainty.

The LOD, based on the number of positive partitions (at least three in each of the three replicates) and required to differentiate a positive sample of low concentration from the blank, was established as 0.5 cp/μL in reaction, which equals 5 cp/μL, approximately in the stock/sample solution (according to the gravimetric dilution). This would be very important if the objective is diagnostic because it will allow taking decisions in advance. After all, the target sequences used would be enough to differentiate a positive from a negative sample at a very low concentration. Nevertheless, this fact confirms one of the most critical characteristics of dPCR, its sensitivity. Theoretically, it will allow the detection of as few as 5 live/dead cells in the sample. However, the sensitivity would be determined by other factors, especially the sample homogeneity and the DNA extraction efficiency.

The specificity test on 16 different *Salmonella* serotypes and other related microorganisms, indicated that these sequences could be used together as potential biomarkers for the detection and quantification of the genus. These five genes are of great importance for detecting *Salmonella*. In total, 329 isolates from environmental and food samples, containing 126 serovars belonging to all subspecies of *Salmonella* were identified using *invA* (Malorny et al., 2007; Kasturi and Drgon, 2017). The *ttr* locus has also been used as an essential molecular marker for reducing false-positive results (Ssemanda et al., 2018; Boer et al., 2019).

**FIGURE 9:**
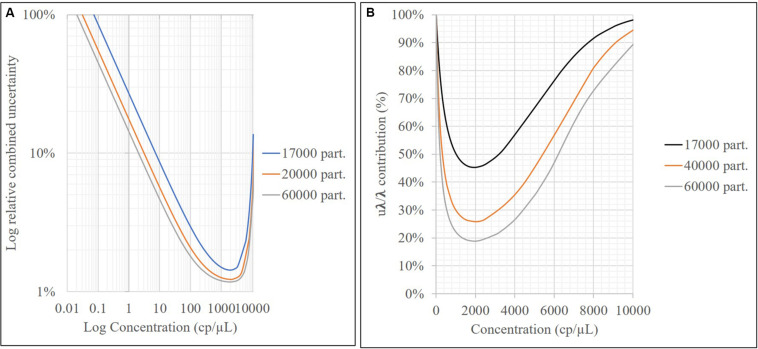
Relative uncertainty associated with the model **(A)** and contribution of *u*λ/λ to mathematical model uncertainty vs. the sample concentration (cp/μL) for different partition numbers **(B)**.

As shown in Figure 4, the relative repeatability standard deviation of the assay varies from 2 to 12% and correlates with the uncertainty according to the mathematical model of the method. Concentration level 2 (800 cp/μL) presents the best precision and also has the lowest uncertainty associated with the model, indicating that it is the best concentration to measure. The mathematical model contribution (Eq. 7) is a function of the number of positive or negative fractions. Subsequently, once the assay has been optimized, it is possible to assign a relative uncertainty component to the sample based on its concentration (Figure 9A). *u*λ/λ is the predominant measurement uncertainty source within the mathematical model, ranging from 50 to 99%. The partition number can be increased to improve this estimation. In the QX200 platform (BioRad, 2018), the maximum partitions per well were 20,000; however, several wells in the Quantasoft^TM^ software can be merged to increase the partition number. This will in turn reduce the *u*λ/λ contribution (Figure 9B), with consequent reduction in combined measurement uncertainty.

Measurement uncertainty contribution was not associated with the correlation between target amplification in the duplex assays. This can be attributed to the primer sequence, which amplifies different regions inside the genome and does not inter-hybridize. Therefore, each primer can function independently.

## Conclusion

After a literature review and *in silico* analysis of the primary target sequences used for the quantification of *Salmonella* spp., five different amplification targets, namely *invA, ttr, hilA, spaQ*, and *siiA*, were selected, which belonged to five genes involved in pathogenicity and metabolism of the bacteria.

According to our results, the method presented here is suitable for estimating the genomic DNA content of *Salmonella* spp. with five different sequences in simplex and duplex form, from 8 to 8,000 cp/μL, with a detection limit of 0.5 copies/μL in the ddPCR reaction, LOQ of 8 cp/μL, precision between 5 and 10%, and combined relative standard measurement uncertainty between 2.0 and 12% over the working interval considering all sources that contribute to it. The most important factor contributing to measurement uncertainty comes from the mathematical model, specifically from the λ factor. To decrease the measurement uncertainty, some wells (at least two) can be merged using the Quantasoft^TM^ software, which will increase the partition number, especially at low concentrations close to the LOQ.

Nevertheless, the unique feature of this assay that enables the detection of five genes instead of one or two ensures higher accuracy and confidence in the results. While a single gene can indicate the presence or absence of *Salmonella* spp., adding other molecular targets provides tools for more reliable quantification, especially for some specific applications such as reference material characterization.

This study contributes to the strengthening of monitoring processes and the increase in competitiveness in the marketing of agricultural industry via developing of metrological assurance tools as validated measurement methods for the identification and quantification of *Salmonella* spp. which is one of the most critical foodborne disease agents. This tool can also be used for the assignment of a reference value for a reference material based on the different sequences evaluated.

## Data Availability Statement

All datasets generated for this study are included in the article/Supplementary Material.

## Author Contributions

CV performed the experiments and wrote the original draft. MC was involved in conceptualization, investigation, and funding acquisition. MA was involved in validation, writing, reviewing, and editing. JL designed the methodology, wrote the original draft, and was involved in project administration. All authors contributed to the article and approved the submitted version.

## Conflict of Interest

The authors declare that the research was conducted in the absence of any commercial or financial relationships that could be construed as a potential conflict of interest.
