# Supplementary material for: Validation of Droplet Digital Polymerase Chain Reaction for Salmonella spp. Quantification
Source: Front Microbiol. 2020 Jul 7;11:1512. doi: 10.3389/fmicb.2020.01512 (PMC7358645; doi:10.3389/fmicb.2020.01512)
Supplement: Supplementary file 1 [file Data_Sheet_1.docx]

**Supplementary information for article “Validation of Droplet Digital Polymerase Chain Reaction for Salmonella spp. Quantification”**

**Supplementary tables**

**Tables S1- S5.** Data of precision experiments performed in three different days at five sample concentration levels with three replicas for each sequence target.

**Table S1**. invA measurement results in ddPCR reaction

| **Level** | **Day** | **Replicate 1** | **Replicate 2** | **Replicate 3** |
| --- | --- | --- | --- | --- |
| 1 | 1 | 8636.62 | 8047.64 | 8090.72 |
|  | 2 | 8803.17 | 8465.04 | 8448.25 |
|  | 3 | 7327.66 | 8176.18 | 7812.01 |
| 2 | 1 | 870.52 | 844.55 | 868.51 |
|  | 2 | 850.63 | 818.72 | 853.32 |
|  | 3 | 839.34 | 811.61 | 833.62 |
| 3 | 1 | 78.24 | 83.99 | 82.62 |
|  | 2 | 75.08 | 68.25 | 73.61 |
|  | 3 | 74.30 | 66.60 | 68.33 |
| 4 | 1 | 7.14 | 7.90 | 7.90 |
|  | 2 | 7.06 | 7.55 | 7.47 |
|  | 3 | 7.13 | 5.06 | 7.31 |
| 5 | 1 | 0.67 | 1.36 | 0.75 |
|  | 2 | 0.66 | 0.57 | 0.94 |
|  | 3 | 0.42 | 1.04 | 1.05 |

**Table S2.** ttr measurement results in ddPCR reaction.

| **Level** | **Day** | **Replicate 1** | **Replicate 2** | **Replicate 3** |
| --- | --- | --- | --- | --- |
| 1 | 1 | 8399.55 | 7884.60 | 8226.53 |
|  | 2 | 7742.07 | 7816.30 | 7790.14 |
|  | 3 | 7312.84 | 7915.42 | 8111.49 |
| 2 | 1 | 761.90 | 765.36 | 792.00 |
|  | 2 | 768.06 | 754.95 | 775.03 |
|  | 3 | 746.47 | 741.44 | 769.48 |
| 3 | 1 | 70.97 | 75.51 | 69.65 |
|  | 2 | 67.65 | 65.24 | 67.30 |
|  | 3 | 61.79 | 63.89 | 64.05 |
| 4 | 1 | 7.62 | 7.28 | 6.66 |
|  | 2 | 6.42 | 5.74 | 5.65 |
|  | 3 | 6.99 | 6.98 | 5.59 |
| 5 | 1 | 0.75 | 0.29 | 0.34 |
|  | 2 | 0.56 | 0.73 | 0.94 |
|  | 3 | 1.27 | 1.30 | 0.49 |

**Table S3**. spaQ measurement results in ddPCR reaction

| **Level** | **Day** | **Replicate 1** | **Replicate 2** | **Replicate 3** |
| --- | --- | --- | --- | --- |
| 1 | 1 | 7974.51 | 8386.08 | 7586.21 |
|  | 2 | 7800.85 | 7868.74 | 7651.99 |
|  | 3 | 6954.66 | 6697.18 | 7109.88 |
| 2 | 1 | 743.61 | 775.78 | 773.39 |
|  | 2 | 784.64 | 804.48 | 836.02 |
|  | 3 | 781.97 | 814.38 | 841.51 |
| 3 | 1 | 82.16 | 85.51 | 87.26 |
|  | 2* | 79.71 |  | 78.64 |
|  | 3 | 78.30 | 80.15 | 85.66 |
| 4 | 1 | 8.31 | 7.85 | 8.43 |
|  | 2 | 9.22 | 11.56 | 8.82 |
|  | 3 | 7.20 | 7.07 | 9.01 |
| 5 | 1 | 1.83 | 1.98 | 1.64 |
|  | 2 | 0.76 | 1.47 | 1.89 |
|  | 3 | 1.62 | 1.40 | 1.96 |

*: There were less than 12000 partitions, this data was not included in the subsequent calculation.

**Table S4**. siiA measurement results in ddPCR reaction

| **Level** | **Day** | **Replicate 1** | **Replicate 2** | **Replicate 3** |
| --- | --- | --- | --- | --- |
| 1 | 1 | 7486.45 | 7603.19 | 7233.45 |
|  | 2 | 7444.69 | 7302.70 | 7297.13 |
|  | 3 | 8012.36 | 7884.80 | 8560.61 |
| 2 | 1 | 822.30 | 817.52 | 814.77 |
|  | 2 | 754.37 | 779.33 | 780.23 |
|  | 3 | 824.65 | 809.76 | 822.79 |
| 3 | 1 | 89.52 | 81.61 | 89.86 |
|  | 2 | 79.71 | 83.20 | 90.74 |
|  | 3 | 83.84 | 83.53 | 86.45 |
| 4 | 1 | 7.65 | 8.87 | 7.15 |
|  | 2 | 7.51 | 9.23 | 10.78 |
|  | 3 | 7.45 | 8.33 | 9.03 |
| 5 | 1 | 1.25 | 1.06 | 1.49 |
|  | 2 | 1.68 | 1.56 | 1.95 |
|  | 3 | 1.64 | 1.11 | 1.89 |

**Table S5**. hilA measurement results in ddPCR reaction

| **Level** | **Day** | **Replicate 1** | **Replicate 2** | **Replicate 3** |
| --- | --- | --- | --- | --- |
| 1 | 1 | 7486.45 | 7803.81 | 7796.76 |
|  | 2 | 7842.03 | 8292.84 | 7792.21 |
|  | 3 | 8401.19 | 8219.88 | 8288.15 |
| 2 | 1 | 875.79 | 866.41 | 866.11 |
|  | 2 | 785.31 | 816.87 | 823.06 |
|  | 3 | 851.14 | 844.49 | 862.22 |
| 3 | 1 | 89.15 | 90.82 | 87.36 |
|  | 2* | 85.41 | 87.01 |  |
|  | 3 | 86.02 | 95.76 | 90.75 |
| 4 | 1 | 10.12 | 10.75 | 9.31 |
|  | 2 | 7.51 | 9.23 | 10.78 |
|  | 3 | 7.72 | 9.59 | 8.96 |
| 5 | 1 | 0.79 | 1.41 | 1.64 |
|  | 2 | 3.52 | 6.09 | 5.69 |
|  | 3 | 4.84 | 5.89 | 5.96 |

*: Third replicate (114,61) was rejected according to Grubb test, at 95 confidence level.

**Table S6.** Simplex vs duplex evaluation (each result is the average of three replicates ± standard deviation).

| **invA** | | **ttr** | | **hilA** | | **siiA** | |
| --- | --- | --- | --- | --- | --- | --- | --- |
| **Simplex** | **Duplex** | **Simplex** | **Duplex** | **Simplex** | **Duplex** | **Simplex** | **Duplex** |
| 8285 ± 958 | 8239 ± 693 | 8024 ± 86 | 7780 ± 416 | 7441 ± 189 | 7792 ± 174 | 8303 ± 92 | 8273 ± 147 |
| 788 ± 51 | 841 ± 19 | 613 ± 27 | 752 ± 15 | 818 ± 4 | 796 ± 12 | 853 ± 9 | 832 ± 14 |
| 77 ± 1 | 72 ± 4 | 69 ± 3 | 63 ± 1.3 | 87 ± 5 | 79 ± 2 | 91 ± 5 | 93 ± 5 |
| 7 ± 0._3_ | 7 ± 0._3_ | *6* ± 0._4_ | *7* ± 0._8_ | 8 ± 0._9_ | 9 ± 1._2_ | 9 ± 1._0_ | 14 ± 1._6_ |

There are not significative differences between simplex and duplex values for every DNA target sequence according to two-sample t-test for unequal variances. Subscripts are indicating second decimal position for the lowest concentration level.

**Table S7.** Relative fraction for every cluster in the duplex amplificación for invA-ttr targets.

| ***invA*+ *ttr*+**  **cluster** | **Relative fraction** | ***invA*+ *ttr*-**  **cluster** | **Relative fraction** | ***invA*- *ttr*+**  **cluster** | **Relative fraction** | ***invA*- *ttr*-**  **cluster** | **Relative fraction** | **Total partitions** | **Ratio**  ***invA/ttr*** |
| --- | --- | --- | --- | --- | --- | --- | --- | --- | --- |
| 42472 | 99,7% | 69 | 0,2% | 47 | 0,1% | 6 | 0,0% | 42594 | 1.05 |
| 10321 | 23,2% | 11808 | 26,6% | 10384 | 23,4% | 11897 | 26,8% | 44410 | 1.1 |
| 150 | 0,3% | 2557 | 5,4% | 2351 | 5,0% | 41969 | 89,2% | 47027 | 1.1 |
| 1 | 0,0% | 305 | 0,6% | 248 | 0,5% | 50526 | 98,9% | 51080 | 1.2 |

**Table S8**. dMIQE checklist

| **ITEM TO CHECK** | **IMPORTANCE** | **Comments** |
| --- | --- | --- |
| **EXPERIMENTAL DESIGN** |  |  |
| Definition of experimental and control groups | **E** | Not applicable |
| Number within each group | **E** | Not applicable |
| Assay carried out by core lab or investigator's lab? | D | Not applicable |
| Power analysis | D | Not applicable |
| **SAMPLE** |  |  |
| Description | **E** | Included in manuscript |
| Volume or mass of sample processed | **E** | Not applicable |
| Microdissection or macrodissection | **E** | Not applicable |
| Processing procedure | **E** | Not applicable |
| If frozen - how and how quickly? | **E** | Not applicable |
| If fixed - with what, how quickly? | **E** | Not applicable |
| Sample storage conditions and duration (especially for FFPE samples) | **E** | Not applicable |
| **NUCLEIC ACID EXTRACTION** |  |  |
| Quantification - instrument/method | **E** | Included in the manuscript |
| Storage conditions: temperature, concentration, duration, buffer | **E** | Included in the manuscript |
| DNA or RNA quantification | **E** | Included in the manuscript |
| Quality/integrity-instrument/method; e.g. RIN/RQI and trace or 3’:5’ | **E** | Not applicable |
| Template structural information | **E** | Not applicable |
| Template modification (digestion, sonication, pre-amplification etc.) | **E** | Not applicable |
| Template treatment (initial heating or chemical denaturation) | **E** | Not applicable |
| Inhibition dilution or spike; | **E** | Not applicable |
| DNA contamination assessment of RNA sample | **E** | Not applicable |
| Details of DNase treatment where performed | **E** | Not applicable |
| Manufacturer of reagents used and catalogue number | D | Not applicable |
| Storage of nucleic acid: temperature, concentration, duration, buffer | **E** | Not applicable |
| **REVERSE TRANSCRIPTION (If necessary)** |  |  |
| cDNA priming method + concentration | **E** | Not applicable |
| One or two step protocol | **E** | Not applicable |
| Amount of RNA used per reaction | **E** | Not applicable |
| Detailed reaction components and conditions | **E** | Not applicable |
| RT efficiency | D | Not applicable |
| Estimated copies measured with and without addition of RT | D | Not applicable |
| Manufacturer of reagents used and catalogue number | D | Not applicable |
| Reaction volume (for two step reverse transcription reaction) | D | Not applicable |
| Storage of cDNA: temperature, concentration, duration, buffer | D | Not applicable |
| **dPCR TARGET INFORMATION** |  |  |
| Sequence accession number | **E** | Not applicable |
| Location of amplicon | D | invA: 2924806 - 2924924  ttr: 1759763 - 1759857  hilA: 2904806 - 2904934  siiA: 4335497 - 43335603  spaQ: 2918074 - 2918186  Reference location in *Salmonella enterica* subsp. *enterica* serovar *Enteritidis* Str EC20122026. GenBank CP0074132 |
| Amplicon length | **E** | Included in manuscript |
| In silico specificity screen (BLAST, etc) | **E** | Not included |
| Pseudogenes, retropseudogenes or other homologs? | D | Not applicable |
| Sequence alignment | D | Not included. |
| Secondary structure analysis of amplicon and GC content | D | Not included in manuscript |
| Location of each primer by exon or intron (if applicable) | **E** | Not applicable |
| Where appropriate, which splice variants are targeted? | **E** | Not applicable |
| **dPCR OLIGONUCLEOTIDES** |  |  |
| Primer sequences and/or amplicon context sequence | **E** | Included in manuscript |
| RTPrimerDB Identification Number | D | Not included |
| Probe sequences | D | Included in manuscript |
| Location and identity of any modifications | **E** | Not applicable |
| Manufacturer of oligonucleotides | D | Included in manuscript |
| Purification method | D | RPC for primers and RP HPLC/dual HPLC for probes |
| **dPCR PROTOCOL** |  |  |
| Complete reaction conditions | **E** | Included in manuscript |
| Reaction volume and amount of RNA/cDNA/DNA | **E** | Included in manuscript |
| Primer, (probe), Mg++ and dNTP concentrations | **E** | Included in manuscript |
| Polymerase identity and concentration | **E** | Included in manuscript |
| Buffer/kit Catalogue No and manufacturer | **E** | Included in manuscript |
| Exact chemical constitution of the buffer | D | Not applicable |
| Additives (SYBR Green I, DMSO, etc.) | **E** | Not applicable |
| Plates/tubes Catalogue No and manufacturer | D | Included in manuscript |
| Complete thermocycling parameters | **E** | Included in manuscript |
| Reaction setup | D | Included in manuscript |
| Gravimetric or volumetric dilutions (manual/robotic) | D | Manual |
| Total PCR reaction volume prepared | D | Included in manuscript |
| Partition number | **E** | 13000 - 19000 |
| Individual partition volume | **E** | Included in manuscript |
| Total volume of the partitions measured (effective reaction size) | **E** | 10.6 – 15.6 uL |
| Partition volume variance/standard deviation | D | Not applicable |
| Comprehensive details and appropriate use of controls | **E** | Included in manuscript |
| Manufacturer of dPCR instrument | **E** | Included in manuscript |
| **dPCR VALIDATION** |  |  |
| Optimisation data for the assay | D | Not included in manuscript |
| Specificity (when measuring rare mutations, pathogen sequences etc.) | **E** | Included in manuscript |
| Limit of detection of calibration control | D | Included in manuscript |
| If multiplexing, comparison with singleplex assays | **E** | Included in manuscript |
| **DATA ANALYSIS** |  |  |
| Average copies per partition (λ or equivalent) | **E** | L1: 8000 cp/uL: 7.01  L2: 800 cp/uL: 6.72E-1  L3: 80 cp/uL: 7.10E-2  L4: 8 cp/uL: 7.31E-3  L5: 1 cp/uL: 7.73E-4 |
| dPCR analysis program (source, version) | **E** | Included in manuscript |
| Outlier identification and disposition | **E** | Included in supplementary information |
| Results of NTCs | **E** | Included in manuscript |
| Examples of positive(s) and negative experimental results as supplemental data | **E** | Included in supplementary information |
| Where appropriate, justification of number and choice of reference genes | **E** | Not applicable |
| Where appropriate, description of normalisation method | **E** | Not applicable |
| Number and concordance of biological replicates | D | Not applicable |
| Number and stage (RT or qPCR) of technical replicates | **E** | Included in manuscript |
| Repeatability (intra-assay variation) | **E** | Included in manuscript |
| Reproducibility (inter-assay/user/lab etc. variation) | D | Included in manuscript |
| Experimental variance or confidence interval | **E** | Included in manuscript |
| Statistical methods used for analysis | **E** | Included in manuscript |
| Data submission using RDML | D | Not included in manuscript |
|  |  |  |

**Supplementary figures**

**Figure S1**. DNA extraction result. A. Agarose electrophoresis (1%, 100V, 60 minutes), 3 µL of DNA stock solution, M: 1 kb DNA ladder (Promega). B: Nanodrop quantification showing absorption spectrum for DNA stock and 1:4 DNA dilution. C: Quantification results.


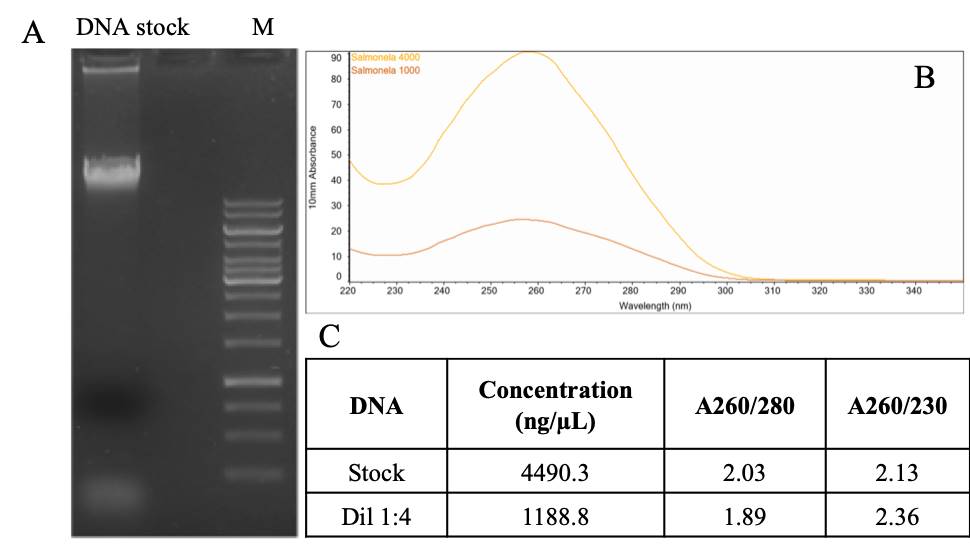


**Figure S2**. Evaluation simplex vs duplex for hilA and siiA targets.


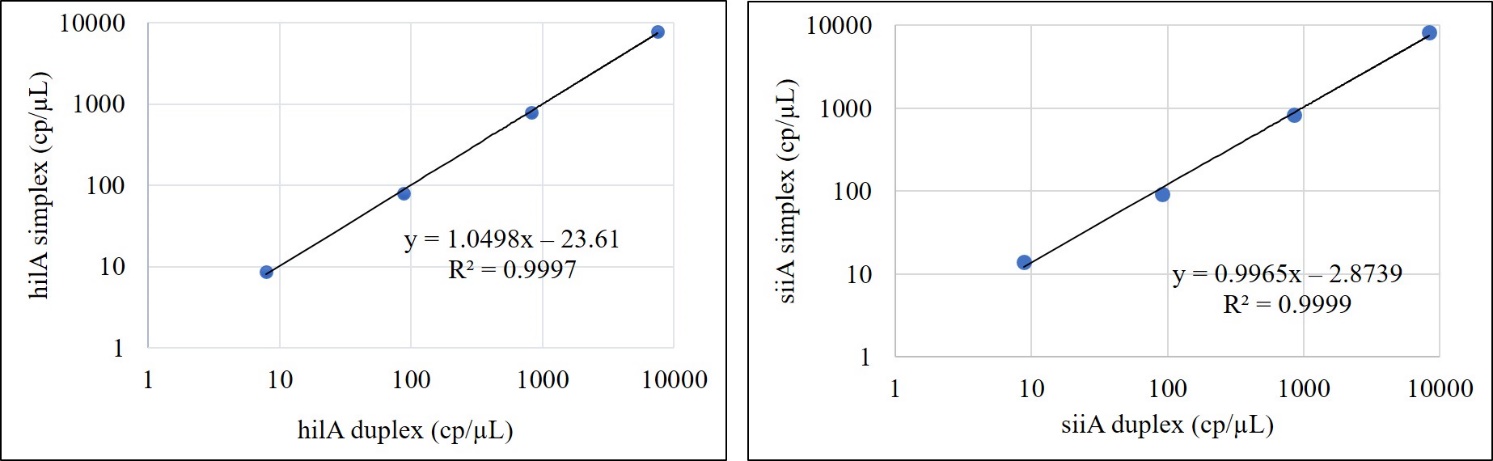


**Figure S3**. Contribution to relative combined standard uncertainty associated to the mathematical model.


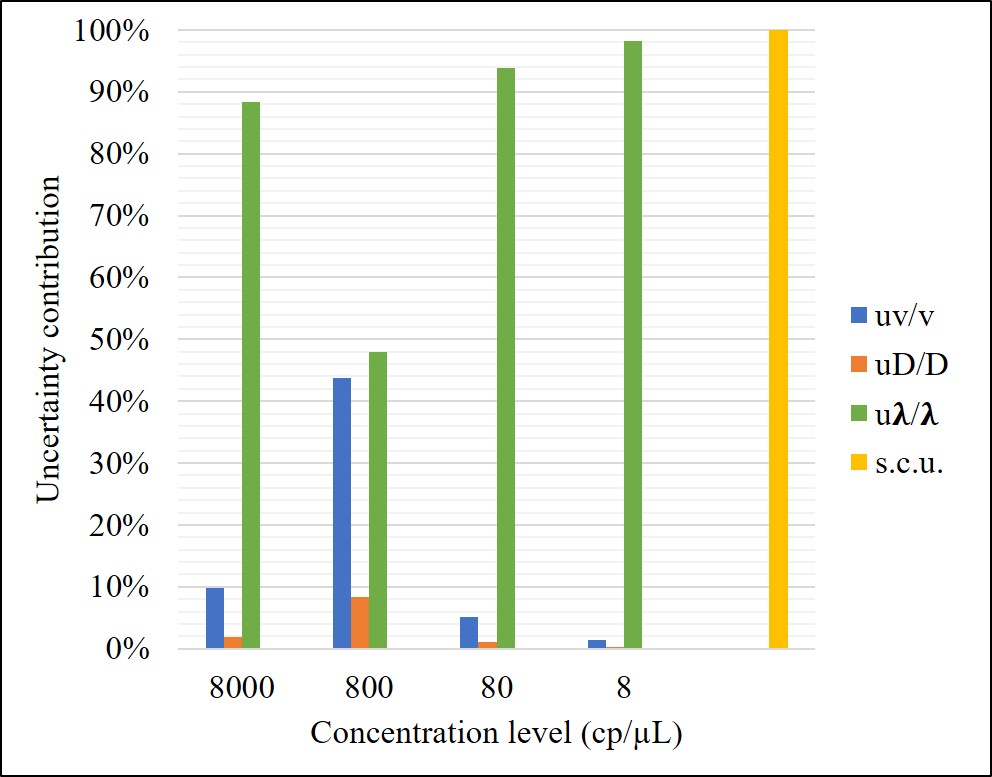


uv/v: relative droplet volume uncertainty, uD/D relative gravimetric dilution uncertainty and uλ/λ relative copies/partition uncertainty factor. s.c.u: Standard combined uncertainty for DNA concentration for every level for invA target.

**Figure S4**. Evaluation of correlation between *siiA* (A) *-hilA* (B) multiplex amplification.

**
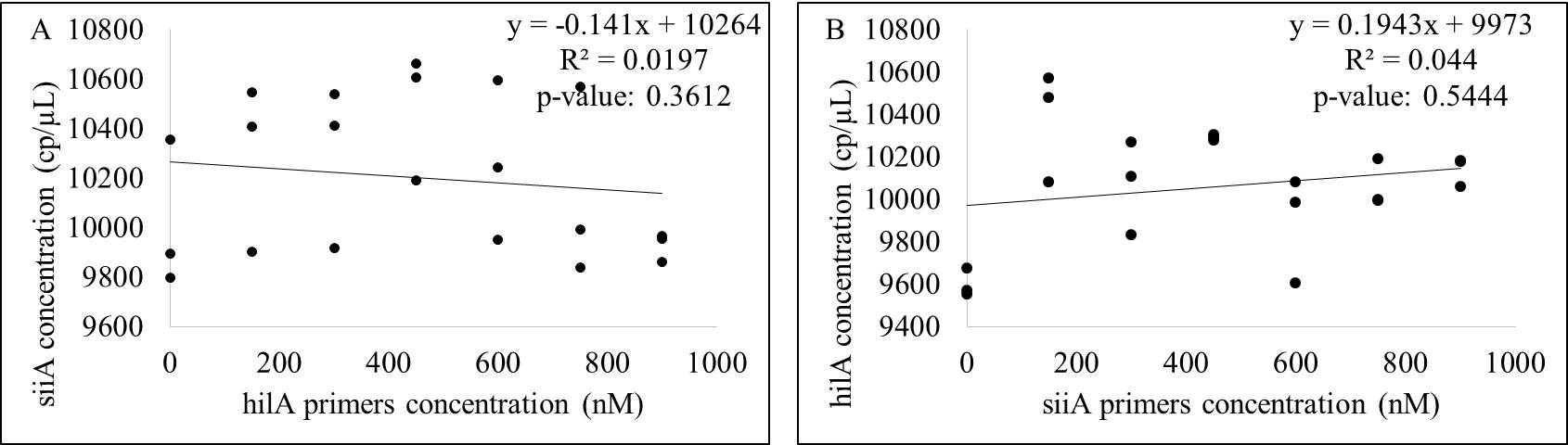
**
